# Pericardial Toxicities Associated With Immune Checkpoint Inhibitors: A Pharmacovigilance Analysis of the FDA Adverse Event Reporting System (FAERS) Database

**DOI:** 10.3389/fphar.2021.663088

**Published:** 2021-07-02

**Authors:** Zhuo Ma, Jie Pei, Ximu Sun, Lihong Liu, Wenchao Lu, Qixiang Guo, Jiayou Lyu, Yuwei Liu, Yuhui Zhang, Zhixia Zhao

**Affiliations:** ^1^ Department of Pharmacy, Beijing Chao-Yang Hospital, Capital Medical University, Beijing, China; ^2^ Department of Pharmacy, Beijing Obstetrics and Gynecology Hospital, Capital Medical University, Beijing, China; ^3^ A.I. Phoenix Technology Co., Ltd., Hong Kong, China; ^4^ Department of Respiratory and Critical Care Medicine, Beijing Institute of Respiratory Medicine, Beijing Chao-Yang Hospital, Capital Medical University, Beijing, China

**Keywords:** immune checkpoint inhibitors, programmed death-1, programmed death-ligand 1, cytotoxic T-lymphocyte-associated protein 4, pericardial toxicities, Food and Drug Administration Adverse Events Reporting System

## Abstract

**Introdution:** Immune checkpoint inhibitors (ICIs) have significantly improved clinical outcomes for a wide range of cancers but can also lead to serious or fatal immune-related adverse events (irAEs). Although ICI-related pericardial toxicities have been reported, the clinical features are not well characterized in real-world studies.

**Objective:** To characterize the main features of ICI-related pericardial toxicities and identify factors associated with death.

**Methods:** Data from January 1, 2011 to March 31, 2020 in the FDA Adverse Event Reporting System database were retrieved for disproportionality analysis. We used the reporting odds ratio and the information component (IC) to evaluate the association between ICIs and pericardial adverse events. Clinical characteristics of patients with ICI-associated pericardial toxicities were collected and compared between fatal and non-fatal groups. The time to onset following different ICI regimens was further investigated.

**Results:** We identified a total of 705 ICI-associated pericardial toxicities which appeared to influence more men (53.90%) than women (36.03%), with a median age of 63 (interquartile range [IQR] 54–69) years. Patients with lung cancer accounted for the largest proportion (55.6%). ICI therapies were detected with pharmacovigilance signals of pericardial toxicities, corresponding to IC_025_ = 2.11 and ROR 4.87 [4.51–5.25]. Nevertheless, there was a lack of association between anti-CTLA-4 and pericardial toxicities. There was no difference in onset time among all ICI regimens. However, TTO of fatal cases (25 days (interquartile range [IQR] 6–70)) occurred statistically earlier than non-fatal cases (42 days (IQR 12–114), *p* = 0.003).

**Conclusion:** ICI monotherapy (PD-1/PD-L1 therapy) and combination therapy can lead to pericardial toxicities that can result in serious outcomes and tend to occur early. Early recognition and management of ICI-related pericardial disorders should attract clinical attention. The findings require further clinical surveillance for the quantification.

## Introduction

Immune checkpoint inhibitors (ICIs) are a novel class of medications for cancer and are revolutionizing the treatment of several major cancers (Postow et al., 2015). The marketed ICIs until recently include programmed death-1 (PD-1) inhibitors (nivolumab, pembrolizumab, cemiplimab), programmed death-ligand 1 (PD-L1) inhibitors (atezolizumab, avelumab, durvalumab), and cytotoxic T-lymphocyte-associated protein 4 (CTLA-4) inhibitors (ipilimumab, tremelimumab) (Ribas and Wolchok, 2018).

Whereas, immune-related adverse events (irAEs) associated with the inhibition of immunologic regulation remains a challenge (Postow et al., 2018). Recently, emerging case reports have raised awareness of cardiotoxicity which maybe potentially a fatal complication associated with immunotherapy (Geisler et al., 2015; Läubli et al., 2015; Johnson et al., 2016). ICI-related pericarditis may occur with or without pericardial effusion and tamponade (Yun et al., 2015; Heinzerling et al., 2016). Perhaps because of inadequate understanding as a form of ICI-related cardiovascular disorders, data on pericardial disease are derived primarily from case reports and clinical trials that may not correctly represent the real world (Borghaei et al., 2015; Kushnir and Wolf, 2017; Atallah et al., 2019; Yamasaki et al., 2019). Moreover, the characteristics, timing, outcomes of ICI-related pericardial toxicities and the factors associated with death are still unknown.

Considering the widespread clinical use of ICIs and the potentially fatal consequences of ICI-associated pericardial toxicities, it is important to identify its clinical manifestations. As such, we conducted a disproportionality analysis to characterize and evaluate pericardial toxicities associated with different ICI regimens. In addition, we further investigated the risk factors and characterized their main features associated with death. All data are based on the Food and Drug Administration Adverse Events Reporting System (FAERS) database.

## Methods

### Data Source

We conducted a retrospective pharmacovigilance study based on the FAERS database (Food and Drug Administration, 2020). The FAERS database collects adverse events (AEs) reports by consumers, health professionals, pharmaceutical manufacturers, and patients from different regions. FAERS data is available to the public. The FAERS data include demographic and administrative information, drug information, reaction information, patient outcomes, the source of the report, therapy start dates and end dates for reported drugs, and indications for use. For this study, ICI data were collected during the period from 2011 Quarter 1 (Q1) to 2020 Quarter 1 (Q1) in the FAERS database.

### Procedures

The report of the FAERS database is coded using preferred terms (PTs) from Medical Dictionary for Regulatory Activities (MedDRA). After literature review and summary of previous studies, we considered the following PTs as related to pericardial disorders: “autoimmune pericarditis [10079058],” “pericarditis [10034484],” “pericarditis adhesive [10034486],” “pericarditis constrictive [10034487],” “pleuropericarditis [10059361],” “cardiac tamponade [10007610],” “pericardial calcification [10057614],” “pericardial disease [10061338],” “pericardial effusion [10034474],” “pericardial fibrosis [10048724],” “pericardial hemorrhage [10034476],” “pericardial mass [10079578],” “pericardial rub [10049759],” “pneumopericardium [10048731],” and “pericardial drainage [10034471].” We selected generic and trade names of ICIs (Table 1) through the drugs@FDA (Food and Drug Administration, 2020). Clinical characteristics (gender, age, reporting time, reporting area, reporter and onset time, etc.) of patients with ICI-associated pericardial toxicities were collected and compared between fatal and non-fatal groups. In addition, we assessed the time to onset (TTO) of pericardial diseases that was caused by different ICIs. We defined the interval from the initiation of the ICI therapy to the start date of the ICI administration as TTO, and excluded the incorrect records.

**TABLE 1:** Summary of FDA approved ICIs.

Generic names	Brand names	Approval year	Target
Nivolumab	Opdivo	2014	PD-1
Pembrolizumab	Keytruda	2014	PD-1
Cemiplimab	Libtayo	2018	PD-1
Atezolizumab	Tecentriq	2016	PD-L1
Avelumab	Bavencio	2017	PD-L1
Durvalumab	Imfinzi	2017	PD-L1
Ipilimumab	Yervoy	2011	CTLA-4
Tremelimumab	—	—	CTLA-4

### Statistical Analysis

We used descriptive statistics to summarize the clinical features of cases. We used the Chi-square test for between-group comparisons of categorical variables. The normally distributed and not normally distributed continuous variables were analyzed using the *t* test and non-parametric tests (the Mann-Whitney test for comparison of two independent samples and the Kruskal-Wallis test for comparison of multiple independent samples), respectively. The statistical significance was determined at *p* < 0.05 with 95% confidence intervals.

The reporting odds ratio (ROR) and Bayesian confidence propagation neural networks of information components (IC) were used to calculate disproportionality (Zhai et al., 2019). When using the full database as a comparator, both IC and ROR were calculated, while when comparing different drug regimen subgroups, only ROR was calculated (Salem et al., 2018). Through the above algorithms, we compared the correlations between pericardial disorders and different ICIs. For IC, a significant signal is considered if an IC_025_ (the lower limit of the IC 95% confidence interval) value greater than zero. For ROR, a significant signal was considered when the lower end of the 95% credibility interval (ROR_025_) exceeded 1, with at least 3 cases. The significant signal indicates an association between a particular drug and the adverse reaction but cannot be interpreted as strictly synonymous with or as a reliable surrogate for the incidence rate. All the analysis was performed with R version 4.0.3.

The statistical formula is as follows to calculate IC, IC = log_2_ ((N_observed_+0.5)/(N_expected_+0.5))

$${\text{N}}_{\text{expected}}=\left(N_{\text{drug}}*N_{\text{event}}\right)/N_{\text{total}}$$

N_expected_: the number of records expected for the drug-adverse reaction combination. N_observed_: the observed number of records for the drug-adverse reaction combination. N_drug_: the total number of records for the drug, regardless of adverse reactions. N_event_: the total number of total records for the adverse reaction, regardless of drug. N_total_: the total number of records in the database.

## Results

### Descriptive Analysis

The FAERS database recorded 87,404 adverse events related to ICIs and 16,862 reports related to pericardial disorders between January 1 2011 and March 31 2020. We screened 705 reports of suspected ICI-related pericardial toxicities and summarized these patients’ clinical characteristics in Table 2. Most cases were reported between 2016 and 2020, reflecting the increasingly clinical application of ICIs in recent years. Most reported cases were male (53.90%; data available in 634/705). The median age was 63 years (interquartile range [IQR] 54–69; data available in 566/705 reports). Most of the cases were from Europe (37.02%), followed by America (31.77%) and Asia (28.51%), and mainly submitted by health-care professionals (78.30%). The median time to event onset was 38 (IQR 12–114; data available in 413/705 reports) days and 44.55% of the adverse events occurred within 30 days. Pericardial adverse events were most frequently reported in lung cancer patients (55.60%). Hospitalization (44.96%) was the most common outcome event, and death occurred in 17.45% of the cases. The largest number of pericardial disorders reports was for nivolumab monotherapy (43.55%), followed by pembrolizumab monotherapy (26.24%).

**TABLE 2:** Characteristics of patients with ICI-associated pericardial diseases sourced from the FAERS database (January 1 2011 to March 31 2020).

Characteristics		Total reports, *n* (%)	Fatal cases, n (%)	Non-fatal cases, n (%)	*p*-value
Total		705	123	580	
Gender	—				NS
	Female	254 (36.03)	45 (36.59)	208 (35.86)	
	Male	380 (53.90)	71 (57.72)	309 (53.28)	
	Unknown or missing	71 (10.07)	7 (5.69)	63 (10.86)	
Age at onset (years)	—				NS
	median (IQR)	63 (54–69)	64 (57–71)	62 (54–69)	
	<18	11 (1.56)	2 (1.63)	9 (1.55)	
	18–64	310 (43.97)	54 (43.90)	254 (43.79)	
	65–84	244 (34.61)	50 (40.65)	194 (33.45)	
	≥85	1 (0.14)	1 (0.81)	0	
	Unknown or missing	139 (19.72)	16 (13.01)	123 (21.21)	
Reporting year	—				NS
	2011	1 (0.14)	0	1 (0.17)	
	2012	0	0	0	
	2013	6 (0.85)	3 (2.44)	3 (0.52)	
	2014	5 (0.71)	2 (1.63)	3 (0.52)	
	2015	29 (4.11)	6 (4.88)	23 (3.97)	
	2016	77 (10.92)	7 (5.69)	70 (12.07)	
	2017	110 (15.60)	19 (15.45)	91 (15.69)	
	2018	201 (28.51)	38 (30.89)	163 (28.10)	
	2019	225 (31.91)	41 (33.33)	182 (31.38)	
	2020 (Q1)	51 (7.23)	7 (5.69)	44 (7.59)	
Reporting region	—				0.029
	Europe	261 (37.02)	48 (39.02)	211 (36.38)	
	America	224 (31.77)	28 (22.76)	196 (33.79)	
	Asia	201 (28.51)	45 (36.59)	156 (26.90)	
	Oceania	15 (2.13)	1 (0.81)	14 (2.41)	
	Africa	0	0	0	
	Unknown or missing	4 (0.57)	1 (0.81)	3 (0.52)	
Indications	—				NS
	Melanoma	61 (8.65)	11 (8.94)	50 (8.62)	
	Lung cancer	392 (55.60)	79 (64.23)	312 (53.79)	
	Hematological cancer and lymphoma	26 (3.69)	2 (1.63)	24 (4.14)	
	Gastrointestinal cancer	28 (3.97)	2 (1.63)	26 (4.48)	
	Head and neck cancer	14 (1.99)	2 (1.63)	12 (2.07)	
	Breast cancer	13 (1.84)	3 (2.44)	10 (1.72)	
	Tumors of female reproductive organs	23 (3.26)	2 (1.63)	20 (3.45)	
	Mesothelioma	10 (1.42)	1 (0.81)	9 (1.55)	
	Non-specifed malignant neoplasm	19 (2.70)	2 (1.63)	17 (2.93)	
	Tumors of urinary system	55 (7.80)	12 (9.76)	43 (7.41)	
	Other indications	17 (2,41)	3 (2.44)	14 (2.41)	
	Unknown or missing	47 (6.67)	4 (3.25)	43 (7.41)	
Outcome	—				
	Death	123 (17.45)	123 (100.00)	0	
	Life-threatening	83 (11.77)	0	83 (14.31)	
	Disability	5 (0.71)	0	5 (0.86)	
	Hospitalization	317 (44.96)	0	317 (54.66)	
	Other serious	175 (24.82)	0	175 (30.17)	
	Unknown or missing	2 (0.28)	-	-	
Reporter TTO (days)	—				NS
	Non-health-care professional	150 (21.28)	34 (27.64)	116 (20.00)	
	Health-care professional	552 (78.30)	89 (72.36)	461 (79.48)	
	Unknown or missing	3 (0.43)	0	3 (0.52)	
	—				0.003
	median (IQR)	38 (12–114)	25 (6–70)	42 (12–114)	
	0–30	184 (26.10)	50 (40.65)	134 (23.10)	
	31–60	69 (9.79)	12 (9.76)	57 (9.83)	
	61–90	42 (5.96)	13 (10.57)	29 (5.00)	
	91–180	55 (7.80)	7 (5.69)	48 (8.28)	
	>180	63 (8.94)	6 (4.88)	56 (9.66)	
	Unknown or missing	292 (41.42)	35 (28.46)	256 (44.14)	
ICI drug as suspected drug	—				NS
	Monotherapy	619 (87.80)	111 (90.24)	506 (87.24)	NS
	Anti-CTLA-4 monotherapy	22 (3.12)	7 (5.69)	15 (2.59)	
	Ipilimumab	22 (3.12)	7 (5.69)	15 (2.59)	
	Tremelimumab	0	0	0	
	Anti-PD-1 monotherapy	496 (70.36)	89 (72.35)	407 (70.18)	
	Pembrolizumab	185 (26.24)	41 (33.33)	144 (24.83)	
	Nivolumab	307 (43.55)	48 (39.02)	259 (44.66)	
	Cemiplimab	4 (0.57)	0	4 (0.69)	
	Anti-PD-L1 monotherapy	101 (14.32)	15 (12.20)	84 (14.48)	
	Atezolizumab	73 (10.35)	13 (10.57)	59 (10.17)	
	Avelumab	4 (0.57)	0	4 (0.69)	
	Durvalumab	24 (3.40)	2 (1.63)	21 (3.62)	
	Combination therapy	86 (12.20)	12 (9.76)	74 (12.76)	NS
	Ipilimumab + nivolumab	79 (11.21)	12 (9.76)	67 (11.55)	
	Ipilimumab + pembrolizumab	4 (0.57)	0	4 (0.69)	
	Tremelimumab + durvalumab	3 (0.43)	0	3 (0.52)	

AbbreviationsICI: immune checkpoint inhibitor; FAERS: Food and Drug Administration's Adverse Event Reporting System; TTO: time to onest.

### Signal Values Associated With Different Immunotherapy Regimens

Generally, ICIs were significantly associated with over-reporting frequencies of pericardial toxicities (ROR 4.87 [4.51–5.25], IC_025_ = 2.11) (Table 3). For monotherapy, the majority of ICI-associated pericardial diseases were reported for anti-PD-1 (70.36%, ROR = 5.67 [5.18–6.21], IC_025_ = 2.31), followed by anti-PD-L1 (14.32%, ROR 5.50 [4.49–6.72], IC_025_ = 2.40), whereas there was a lack of association between anti-CTLA-4 and pericardial adverse events. The most common combination therapy was nivolumab plus ipilimumab (79 cases), with a significant pericardial events signal (ROR 4.20 [3.37–5.25], IC025 = 1.69). As for the combination of pembrolizumab plus ipilimumab, and durvalumab plus tremelimumab, there were only 4 and 3 cases, respectively. Further analysis showed that combination therapy was not associated with a higher risk of pericardial toxicities compared with monotherapy (ROR 0.91 [0.73–1.14].

**TABLE 3:** Associations of different ICI regimens with pericardial diseases.

Strategy	Drug	N	ROR	ROR_025_	ROR_975_	IC	IC_0.25_
Total	Total ICIs	705	4.87	4.51	5.25	2.22	2.11
Monotherapy	Anti-PD-1	496	5.67	5.18	6.21	2.45	2.31
	Nivolumab	307	5.45	4.87	6.11	2.40	2.24
	Pembrolizumab	185	5.26	4.54	6.08	2.35	2.14
	Cemiplimab	4	6.34	2.36	16.99	1.82	0.38
	Anti-PD-L1	101	5.50	4.49	6.72	2.40	2.10
	Atezolizumab	73	6.05	4.81	7.63	2.52	2.18
	Avelumab	4	2.02	0.76	5.40	0.69	-0.75
	Durvalumab	24	5.01	3.35	7.48	2.17	1.58
	Anti-CTLA-4	22	1.36	0.89	2.06	0.40	-0.22
	Tremelimumab	0	—				
	Ipilimumab	22	1.27	0.84	1.93	0.31	-0.31
Anti-PD-1vs anti-ctla-4	—	—	4.07	2.66	6.25	—	—
Anti-PD-L1vs anti-ctla-4			4.03	2.54	6.42		
Anti-PD-1 vs anti-PD-L1			1.01	0.81	1.26		
Combination therapy			—				
—	Ipilimumab + nivolumab	79	4.20	3.37	5.25	2.02	1.69
	Ipilimumab + pembrolizumab	4	5.09	1.90	12.61	1.64	0.19
	Tremelimumab + durvalumab	3	6.25	2.00	19.49	1.62	-0.05
Combination vs monotherapy therapy	—	—	0.91	0.73	1.14	—	

AbbreviationsN: number of records; ROR025: the lower end of the 95% confidence interval of ROR. ROR975: the upper end of the 95% confidence interval of IC; IC025: the lower end of the 95% confidence interval of IC.

### Analysis of Fatal and Non-Fatal Cases

As shown in Table 2, no significant differences were found in gender, age, reporter type, outcome, indication and year of reporting for fatal vs. non-fatal cases. Use of ICIs combination vs. ICI monotherapy and type of monotherapy (anti-PD-1, anti-PD-L1 and anti-CTLA4) were similar in fatal vs. non-fatal ICI-related pericardial toxicity cases. In the two most common indications, the fatality was higher in patients with lung cancer compared to melanoma, with no significant difference (20.15 vs 18.03%). Notably, time to onset was statistically different between two groups of cases. The fatal cases had a shorter TTO than non-fatal cases (median 25 days (IQR 6–70 days) vs. 42 days (IQR12–114), respectively; *p* = 0.003). Besides, there was a statistical difference between fatal and non-fatal cases in the reporting region (*p* = 0.029), with the highest percentage of reported deaths (22.39%, 45/201) occurring in Asia.

### Time to Onset of Immune Checkpoint Inhibitors-Associated Pericardial Disease

**FIGURE 1:**
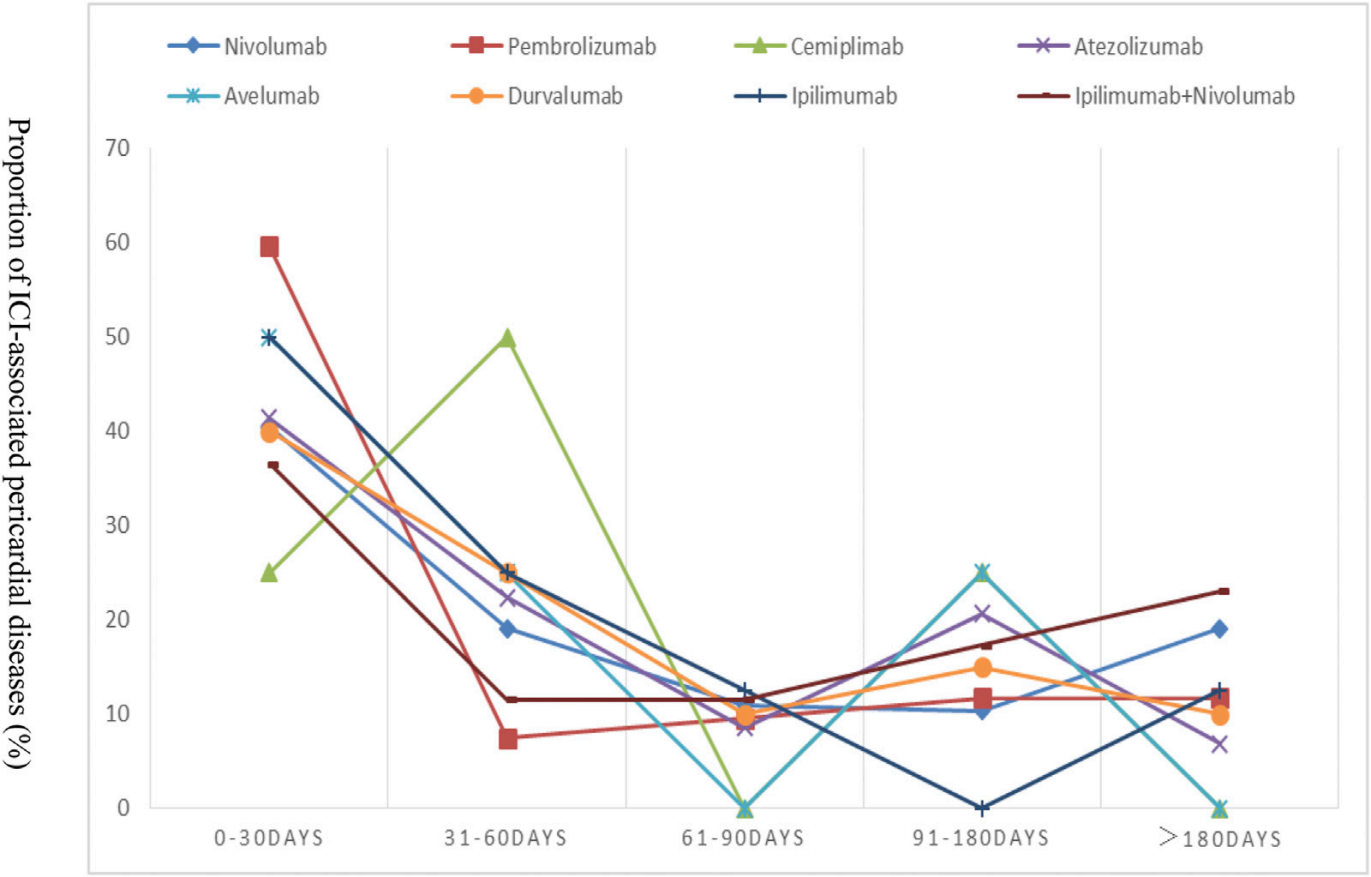
Time to event onset of pericardial adverse events following immune checkpoint inhibitor regimens.

The TTO following each ICI regimen is shown in Figure 1. We found no difference in onset time among ICI monoregimens (*p* = 0.350). The median time to onset was 31 (IQR 19–53) days for ipilimumab, 21 (IQR 7–81) days for pembrolizumab, 42 (IQR 14–127) days for nivolumab, 42 (IQR 39–60) days for cemiplimab, 37 (IQR 11–101) days for atezolizumab, 32 (IQR 18–58) days for avelumab, and 52 (IQR12-92) days for durvalumab, respectively. In addition, patients treated with a combination regimen didn’t appear to have earlier onset compared with those receiving monotherapy alone. On the contrary, TTO of ipilimumab plus nivolumab combination therapy was later than that of ipilimumab or nivolumab monotherapy alone.

## Discussion

To our knowledge, this is the most comprehensive pharmacovigilance study on pericardial events associated with ICIs based on the FAERS database. We provided a more accurate description and characterization of ICI-associated pericardial toxicities to date.

Our study detected a significant signal between ICI treatment and pericardial toxicities. We included 705 cases of ICI-associated pericardial diseases, the largest such collection of cases to date, suggesting that pericardial-irAEs under ICIs appear less rare than initially thought (Saade et al., 2019). The increased reporting over time was mainly due to the wider use of ICIs and increased awareness of healthcare professionals for post-marketing surveillance. Notably, the signal identified for pericardial toxicities might be amplified by over-reporting, since pericardial disorders are common complications of some malignancies (Imazio et al., 2005). It is vital to distinguish between tumor complications and irAEs in clinical practice because the definitive interruption of ICIs for patients may be challenged. The diagnosis of ICI-related pericardial events remains an excluded diagnosis, requiring the exclusion of other identified causes (Saade et al., 2019).

ICI-associated pericardial diseases seemed to predominately affect men (60%, 380/634), which was consistent with the findings of Salem et al. using the World Health Organization (WHO) global database vigibase (Salem et al., 2018). However, it should be noted that women were often excluded from the clinical trials and not recommended to use ICIs in real world settings due to a higher risk of autoimmune diseases (Conforti et al., 2018; Hu et al., 2019). And the preponderance of men in ICI-associated pericardial diseases may be due to the fact that melanoma or lung cancer is more common in men than in women (Henley et al., 2014; Salem et al., 2018). Therefore, further studies are needed to provide the true incidence of ICI-associated pericardial diseases in men and women.

This study showed that ICI-associated pericardial disorders were over-reported for anti-PD-1/PD-L1 vs. anti-CTLA-4 monotherapy (ROR: 4.07 [2.66–6.25] and 4.03 [2.54–6.42], respectively). In addition, there was no increased risk with combination therapy (anti-CTLA-4 plus anti-PD-1 or anti-PD-L1) relative to monotherapy (anti-PD-1 or anti-PD-L1 alone) (ROR: 0.91 [0.73–1.14]). Anti-CTLA-4 agents were surprisingly not associated with risk of reporting pericardial toxicities (ROR: 1.36 [0.89–2.06]), in contrast to previous studies showing that anti-CTLA-4 was associated with over-reporting frequencies of myocarditis (Fan et al., 2019). The low number of events observed of CTLA-4 highlights the need to study differences between these classes. Moreover, comparisons with different ICI classes could be confounded by disease-specific effects. ICIs are approved for different indications, such as melanoma patients largely comprising the anti-CTLA-4 group, and the difference of approved indications may also account for the different risk shown in our study. Pericardial diseases were, indeed, more frequent in patients with lung cancer (55.6%). The disease-specific effects of lung cancer in our study are unclear. One reasonable hypothesis is the proposed synergistic effects of radiation and immunotherapies which may be at play in lung cancer patients. Radiation’s possible ability to prime an endogenous antigen-specific immune response has been used a rationale for combining radiation and immunotherapies for synergistic effects (Sharabi et al., 2015). In this regard, it is plausible that patients with cancer who receive ICI following irradiation to the thoracic area may be more prone to pericardial disorders, exposing potential shared antigens to T-cell recognition (Salem et al., 2018). However, further reliable data are needed to definitively rule out an association between anti-CTLA-4 and pericardial toxicities.

Our studies suggested ICI-associated pericardial diseases occurred early after the initiation of ICIs, with no significant difference between different ICI regimens. Cardiac disorders were reported to occur between 2 and 17 weeks after initiation ICI therapy (Wang et al., 2017; Oristrell et al., 2018). The median time from start of ICIs to presentation in this study was 38 days (12–114) and the majority of cases were detected within the first 3 months which indicated the importance of cardiac monitoring during the higher-risk time window of 90 days. However, no earlier onset of pericardial diseases was found with the nivolumab plus ipilimumab strategy than with nivolumab alone. This finding was inconsistent with those of previously published cases of ICI-associated cardiotoxicity, who found that cardiotoxicity occurred earlier when two ICIs were combined (Zhou et al., 2019).

The risk of ICI-induced adverse events resulting in death was studied in detail. Use of ICI combination vs. ICI monotherapy and type of monotherapy were similar in fatal vs. non-fatal cases. However, it was worth noting that anti-CTLA-4 had the lowest number but the highest mortality (31.82%, 7/22). We observed a significant difference in the fatality rates in different reported regions, with Asia being the highest (22.39%, 45/201) and Oceania the lowest (6.67%, 1/15). Bhandari et al. found no racial difference in the incidence and severity of irAEs between whites and African Americans although autoimmune diseases generally exhibit racial differences (Bhandari et al., 2020). Thompson et al. observed similar cutaneous immune-related adverse events (cirAEs) morphologies and severities among white and non-white patients but white patients were twice as likely to be diagnosed with a cirAE (Thompson et al., 2020). These two studies were all conducted in the United States. The association of race with the incidence and severity of irAEs secondary to ICIs was still unclear, especially outside the United States. The difference in fatality rates among reported regions found here could not prove a causal relationship between race and death. However, it is suggested that the Asian population should pay particular attention to the risk of pericardial disease caused by ICIs due to its high mortality rate. Notably, we observed an earlier onset of ICI-related pericardial toxicities in fatal cases compared to non-fatal cases, highlighting the need for understanding the biological mechanisms and identifying risk factors that could assist in the diagnosis and effective management of these patients. Overall, more data are needed to identify patient-specific risk factors for this toxicity.

Our study has several limitations. Firstly, FAERS is a spontaneous reporting system with a reporting bias (e.g., underreporting and selective reporting) and lots of missing data. It is difficult to control confounding factors such as age, history of cardiovascular disease, comorbidities or other factors that could influence the risk of cardiotoxicity. Therefore, a causal relationship cannot be directly proven. Secondly, a spontaneous reporting system is only used for qualitative research. The collected data cannot be used to quantify the adverse reaction signals on the basis of the total number of adverse reactions. The signal strength between a particular drug and the reaction was only used as a qualitative indicator. Safety reports do not provide detailed information of patients exposed to the drug without AEs (Ceschi et al., 2020). Moreover, the identification and reporting of adverse events are not rigorously regulated. There are missing reports and duplicate reports. Therefore, FAERS data cannot be used to calculate the incidence of an adverse event. Thirdly, database reporting is voluntary and thus the quality might be variable. Like other pharmacovigilance studies, this study allows for signal detection in a large population, which will require further researches to validate the results.

## Conclusion

This study explored reports of pericardial adverse events associated with the use of ICIs in the FAERS database. A total of 705 reports were retrieved, more than half of which were patients with lung cancer. Our study shows that ICI-related pericardial toxicities commence early during treatment course and can result in serious outcomes. These events should be considered in patient care and clinical trial design. Further studies are needed to address the mechanisms underlying ICI pericardial toxicities associated with ICIs and assess the causality of the cases to draw conclusions on the strength of the relationships.

## Data Availability

The original contributions presented in the study are included in the article/Supplementary Material, further inquiries can be directed to the corresponding authors.

## References

[B1] Atallah-Yunes S. A. Kadado A. J. Soe M. H. (2019). Pericardial Effusion Due to Pembrolizumab-Induced Immunotoxicity: A Case Report and Literature Review. Current Problems Cancer 43 (5), 504–510. 10.1016/j.currproblcancer.2019.01.001 30685067

[B2] Bhandari S. Kumar R. Nice L. Cheema A. Kloecker G. H. (2020). Racial Differences in Development of Immune-Related Adverse Events. Jco 38 (15_), e15148. 10.1200/jco.2020.38.15_suppl.e15148

[B3] Borghaei H. Paz-Ares L. Horn L. Spigel D. R. Steins M. Ready N. E. (2015). Nivolumab versus Docetaxel in Advanced Nonsquamous Non-small-Cell Lung Cancer. N. Engl. J. Med. 373 (17), 1627–1639. 10.1056/NEJMoa1507643 26412456 PMC5705936

[B4] Ceschi A. Noseda R. Palin K. Verhamme K. (2020). Immune Checkpoint Inhibitor-Related Cytokine Release Syndrome: Analysis of WHO Global Pharmacovigilance Database. Front. Pharmacol. 11, 557. 10.3389/fphar.2020.00557 32425791 PMC7212758

[B5] Conforti F. Pala L. Bagnardi V. De Pas T. Martinetti M. Viale G. (2018). Cancer Immunotherapy Efficacy and Patients' Sex: a Systematic Review and Meta-Analysis. Lancet Oncol. 19 (6), 737–746. 10.1016/S1470-2045(18)30261-4 29778737

[B6] Fan Q. Hu Y. Yang C. Zhao B. (2019). Myocarditis Following the Use of Different Immune Checkpoint Inhibitor Regimens: A Real-World Analysis of post-marketing Surveillance Data. Int. Immunopharmacology 76, 105866. 10.1016/j.intimp.2019.105866 31491729

[B7] Geisler B. P. Raad R. A. Esaian D. Sharon E. Schwartz D. R. (2015). Apical Ballooning and Cardiomyopathy in a Melanoma Patient Treated with Ipilimumab: a Case of Takotsubo-like Syndrome. J. Immunotherapy Cancer 3, 4. 10.1186/s40425-015-0048-2 PMC4335413 25705383

[B8] Heinzerling L. Ott P. A. Hodi F. S. Husain A. N. Tajmir-Riahi A. Tawbi H. (2016). Cardiotoxicity Associated with CTLA4 and PD1 Blocking Immunotherapy. J. Immunotherapy Cancer 4 (1), 50. 10.1186/s40425-016-0152-y PMC4986340 27532025

[B9] Henley S. J. Richards T. B. Underwood J. M. Eheman C. R. Plescia M. McAfee T. A (2014). Lung Cancer Incidence Trends Among Men and women--United States, 2005-2009. MMWR Morb Mortal Wkly Rep. 63 (1), 1–5. 24402465 PMC5779336

[B10] Hu J.-R. Florido R. Lipson E. J. Naidoo J. Ardehali R. Tocchetti C. G. (2019). Cardiovascular Toxicities Associated with Immune Checkpoint Inhibitors. Cardiovasc. Res. 115 (5), 854–868. 10.1093/cvr/cvz026 30715219 PMC6452314

[B11] Imazio M. Demichelis B. Parrini I. Favro E. Beqaraj F. Cecchi E. (2005). Relation of Acute Pericardial Disease to Malignancy. Am. J. Cardiol. 95 (11), 1393–1394. 10.1016/j.amjcard.2005.01.094 15904655

[B12] Johnson D. B. Balko J. M. Compton M. L. Chalkias S. Gorham J. Xu Y. (2016). Fulminant Myocarditis with Combination Immune Checkpoint Blockade. N. Engl. J. Med. 375, 1749–1755. 10.1056/NEJMoa1609214 27806233 PMC5247797

[B13] Kushnir I. Wolf I. (2017). Nivolumab-Induced Pericardial Tamponade: A Case Report and Discussion. Cardiology 136 (1), 49–51. 10.1159/000447053 27554835

[B14] Läubli H. Balmelli C. Bossard M. Pfister O. Glatz K. Zippelius A. (2015). Acute Heart Failure Due to Autoimmune Myocarditis under Pembrolizumab Treatment for Metastatic Melanoma. J. Immunotherapy Cancer 3, 11. 10.1186/s40425-015-0057-1 PMC4404586 25901283

[B15] Oristrell G. Bañeras J. Ros J. Muñoz E. (2018). Cardiac Tamponade and Adrenal Insufficiency Due to Pembrolizumab: a Case Report. Eur. Heart J. Case Rep. 2 (2), yty038. 10.1093/ehjcr/yty038 31020118 PMC6177032

[B16] Postow M. A. Callahan M. K. Wolchok J. D. (2015). Immune Checkpoint Blockade in Cancer Therapy. Jco 33, 1974–1982. 10.1200/JCO.2014.59.4358 PMC4980573 25605845

[B17] Postow M. A. Sidlow R. Hellmann M. D. (2018). Immune-related Adverse Events Associated with Immune Checkpoint Blockade. N. Engl. J. Med. 378 (2), 158–168. 10.1056/NEJMra1703481 29320654

[B18] Ribas A. Wolchok J. D. (2018). Cancer Immunotherapy Using Checkpoint Blockade. Science 359, 1350–1355. 10.1126/science.aar4060 29567705 PMC7391259

[B19] Saade A. Mansuet-Lupo A. Arrondeau J. Thibault C. Mirabel M. Goldwasser F. (2019). Pericardial Effusion under Nivolumab: Case-Reports and Review of the Literature. J. Immunotherapy Cancer 7 (1), 266. 10.1186/s40425-019-0760-4 PMC6798500 31627742

[B20] Salem J.-E. Manouchehri A. Moey M. Lebrun-Vignes B. Bastarache L. Pariente A. (2018). Cardiovascular Toxicities Associated with Immune Checkpoint Inhibitors: an Observational, Retrospective, Pharmacovigilance Study. Lancet Oncol. 19 (12), 1579–1589. 10.1016/S1470-2045(18)30608-9 30442497 PMC6287923

[B21] Sharabi A. B. Lim M. DeWeese T. L. Drake C. G. (2015). Radiation and Checkpoint Blockade Immunotherapy: Radiosensitisation and Potential Mechanisms of Synergy. Lancet Oncol. 16 (13), e498–e509. 10.1016/S1470-2045(15)00007-8 26433823

[B22] Thompson L. L. Pan C. X. Chang M. S. Krasnow N. A. Blum A. E. Chen S. T. (2021). Impact of Ethnicity on the Diagnosis and Management of Cutaneous Toxicities from Immune Checkpoint Inhibitors. J. Am. Acad. Dermatol. 84, 851–854. 10.1016/j.jaad.2020.09.096 33080345

[B23] U.S. Food and Drug Administration (2020a). Questions and Answers on FDA's Adverse Event Reporting System (FAERS). http://www.fda.gov/Drugs/GuidanceComplianceRegulatoryInformation/Surveillance/AdverseDrugEffects (Accessed July 30, 2020).

[B24] U.S. Food and Drug Administration (2020b). Find Drugs and Conditions. https://www.drugs.com. (Accessed July 29, 2020).

[B25] Wang D. Y. Okoye G. D. Neilan T. G. Johnson D. B. Moslehi J. J. (2017). Cardiovascular Toxicities Associated with Cancer Immunotherapies. Curr. Cardiol. Rep. 19 (3), 21. 10.1007/s11886-017-0835-0 28220466 PMC10176498

[B26] Yamasaki M. Daido W. Saito N. Funaishi K. Okada T. Kawamoto K. (2019). Pericardial Effusion with Tamponade in Lung Cancer Patients during Treatment with Nivolumab: A Report of Two Cases. Front. Oncol. 9, 4. 10.3389/fonc.2019.00004 30723699 PMC6349695

[B27] Yun S. Vincelette N. D. Mansour I. Hariri D. Motamed S. (2015). Late Onset Ipilimumab-Induced Pericarditis and Pericardial Effusion: A Rare but Life Threatening Complication. Case Rep. Oncological Med. 2015, 1–5. 10.1155/2015/794842 PMC4396732 25918658

[B28] Zhai Y. Ye X. Hu F. Xu J. Guo X. Zhuang Y. (2019). Endocrine Toxicity of Immune Checkpoint Inhibitors: a Real-World Study Leveraging US Food and Drug Administration Adverse Events Reporting System. J. Immunotherapy Cancer 7 (1), 286. 10.1186/s40425-019-0754-2 PMC6836403 31694698

[B29] Zhou Y.-W. Zhu Y.-J. Wang M.-N. Xie Y. Chen C.-Y. Zhang T. (2019). Immune Checkpoint Inhibitor-Associated Cardiotoxicity: Current Understanding on its Mechanism, Diagnosis and Management. Front. Pharmacol. 10, 1350. 10.3389/fphar.2019.01350 31849640 PMC6897286

